# Titin kinase is an inactive pseudokinase scaffold that supports MuRF1 recruitment to the sarcomeric M-line

**DOI:** 10.1098/rsob.140041

**Published:** 2014-05-21

**Authors:** Julijus Bogomolovas, Alexander Gasch, Felix Simkovic, Daniel J. Rigden, Siegfried Labeit, Olga Mayans

**Affiliations:** 1 Department of Integrative Pathophysiology, Medical Faculty Mannheim, University of Heidelberg, Mannheim 68167, Germany; 2 Institute of Integrative Biology, Biosciences Building, University of Liverpool, Crown St., Liverpool L69 7ZB, UK

**Keywords:** titin, pseudokinase, mutagenesis, phosphorylation assay

## Abstract

Striated muscle tissues undergo adaptive remodelling in response to mechanical load. This process involves the myofilament titin and, specifically, its kinase domain (TK; titin kinase) that translates mechanical signals into regulatory pathways of gene expression in the myofibril. TK mechanosensing appears mediated by a C-terminal regulatory tail (CRD) that sterically inhibits its active site. Allegedly, stretch-induced unfolding of this tail during muscle function releases TK inhibition and leads to its catalytic activation. However, the cellular pathway of TK is poorly understood and substrates proposed to date remain controversial. TK's best-established substrate is Tcap, a small structural protein of the Z-disc believed to link TK to myofibrillogenesis. Here, we show that TK is a pseudokinase with undetectable levels of catalysis and, therefore, that Tcap is not its substrate. Inactivity is the result of two atypical residues in TK's active site, M34 and E147, that do not appear compatible with canonical kinase patterns. While not mediating stretch-dependent phospho-transfers, TK binds the E3 ubiquitin ligase MuRF1 that promotes sarcomeric ubiquitination in a stress-induced manner. Given previous evidence of MuRF2 interaction, we propose that the cellular role of TK is to act as a conformationally regulated scaffold that functionally couples the ubiquitin ligases MuRF1 and MuRF2, thereby coordinating muscle-specific ubiquitination pathways and myofibril trophicity. Finally, we suggest that an evolutionary dichotomy of kinases/pseudokinases has occurred in TK-like kinases, where invertebrate members are active enzymes but vertebrate counterparts perform their signalling function as pseudokinase scaffolds.

## 2. Introduction

The giant protein titin (3–4.2 MDa depending on spliceoform) is believed to orchestrate the response of muscle to mechanical and metabolic stress. Single titin molecules span entire half-sarcomeres (from Z-disc to M-lines, more than 1 µm in length) and contain strain-compliant elements, forming an elastic lattice within the cytoskeleton of acto-myosin motors [1]. Titin binds an extensive range of myofibrillar proteins, including Tcap/telethonin (which has cardio-protective roles) [2], transcriptional regulators [3,4], and remodelling factors such as calpain proteases [5,6] and E3 ubiquitin ligases [7,8]. These proteins link titin to the regulation of the membrane potential [9], and to protein turnover and gene expression processes in the sarcomere [10]. Taken together, the elastic character of the titin chain and its multiple scaffolding interactions make it an optimal platform to integrate cellular responses to force sensing in muscle [1].

The kinase domain near the C-terminus of titin (TK) in the sarcomeric M-line plays an important role in mechanotransduction. TK binds a protein complex formed by the autophagosomal receptors nbr1 and p62, and the E3 ubiquitin ligase MuRF2 [10]. A force-dependent regulation of this complex was inferred by inducing the beating arrest of cardiomyocytes under hyperkalemic depolarization [10]. The arrest induced the disassembly of the TK signalosome and the subsequent translocation of MuRF2 to the cell nucleus. There, MuRF2 appeared to block the anabolic action of the serum response transcription factor. Intriguingly, a docking site for the E3 ubiquitin ligase MuRF1 (a close homologue of MuRF2) has been mapped to a tandem of Ig domains in titin preceding TK [7,8]. MuRF1 is strongly upregulated by atrophic stimuli such as immobilization, denervation, nutritional deprivation, aging and disease (e.g. cancer, sepsis and renal failure), being an important mediator of muscle waste [11,12]. However, a functional interrelation between TK/MuRF2 and the vicinal MuRF1 has not been found, leaving unclear how this M-line node in titin is coordinated as a whole.

The importance of TK for muscle physiology is demonstrated by the fact that its genetic defect results in life-threatening myopathies in patients [10]. However, the signalling pathway of TK in the cell remains elusive, and efforts to identify its sarcomeric substrates have yielded few candidates. Three sarcomeric proteins have been proposed as phosphorylation substrates of TK: Tcap [13], nbr1 and p62 [10]. Nbr1 and p62 elicit weak catalysis *in vitro* and their phosphorylation is seemingly unrelated to their function in the TK-signalosome [10]. By contrast, Tcap is subject to notable levels of phosphorylation, being TK's best-established substrate. Tcap anchors titin in the Z-disk, cross-linking the N-termini of two neighbouring titin molecules [14], and further connects titin to MLP- and minK-associated stretch signalling [2,9]. Tcap was initially identified as a TK substrate in differentiating myotubes and its modification was regarded as pointing to TK roles in the regulation of myofibrillogenesis [13].

Despite the scarcity of candidate substrates, all proposed roles of TK in cell signalling assume a kinase activity, where phospho-transfer occurs in a stretch-regulated fashion. The crystal structure of TK [13] appeared to suggest that the kinase was inhibited by a CRD that folds against the catalytic core, binding deeply into the ATP-binding pocket (electronic supplementary material, figure S1). In addition, the presumed catalytic aspartate at the active site was blocked by an interaction with a tyrosine residue, Y170, from the P+1 loop. For TK activation, the steric blockage imposed by both Y170 and CRD would need to be removed. Early studies [13] indicated that Y170 inhibition is released by phosphorylation by a developmentally regulated kinase, but the latter has remained unidentified. More uncertain is the mechanism of CRD displacement as biochemical activators that bind this tail are yet to be identified. However, based on atomic force microscopy data and molecular dynamics simulations, a mechanoactivation hypothesis has been recently proposed [15–17]. This hypothesis postulates that cytoskeletal stretch during myofibril function pulls the CRD from the active site, freeing the kinase to adopt a catalytically active conformation. This mechanosensory mechanism agrees with the proposed involvement of TK in stretch-activated pathways in muscle [10].

For future progress in understanding TK function, the interplay between its scaffolding, catalytic and mechanosensory processes must be resolved. In this study, we focused on elucidating the regulation of TK phospho-transfer, applying structural and catalytic approaches. Our data reveal that TK is a pseudokinase with non-detectable catalytic output. Instead, it is a high-affinity binding locus for MuRF1, constituting a cross-talk node for the MuRF1 and MuRF2 ubiquitin ligases. This result points to a new direction in understanding the role of TK in muscle signalling, where scaffolding and not kinase activity is to take centre stage.

## 3. Results

### 3.1. Preparations from insect cells contain a contaminant Tcap phosphorylating activity

In an attempt to characterize the catalytic profile of TK, we first set out to study its phosho-transfer activity on the Tcap substrate. For this, we assayed three TK variants (comprising catalytic kinase domain and CRD) expressed in Sf21 insect cells: wild-type TK, the activated phosphomimic TK^Y170E^ and the constitutively inactive TK^K36L^ (mutated residues illustrated in the electronic supplementary material, figure S1**)**. In TK^Y170E^, the inhibition of the catalytic aspartate by the tyrosine residue in the P+1 loop is removed and the sample was proposed to be constitutively active [10,13,18]. In TK^K36L^, the highly conserved lysine residue involved in the coordination and catalysis of ATP is mutated into an unreactive leucine group that abolishes phospho-transfer. Mutation of this lysine residue into, for example, alanine, histidine, methionine or isoleucine, is an established method to eliminate catalytic activity (e.g. [19,20]). Although mutation to alanine is most common, in TK this exchange resulted in certain structural instability. Using FoldX [21] and differential scanning fluorimetry to measure sample melting curves, we identified leucine to be well tolerated by the TK fold (electronic supplementary material, §S2). Thus, TK^K36L^ was used as inactive variant throughout this work.

**Figure 1.**
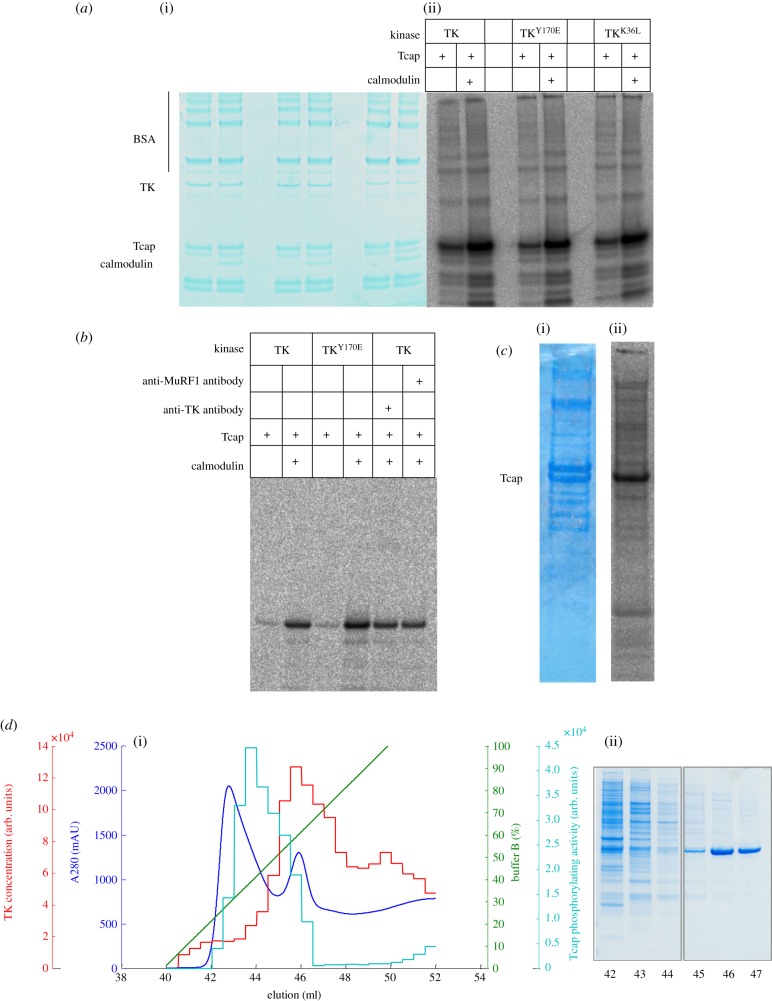
Tcap phosphorylation assays using TK preparations from insect cells. (*a*) Preparations of wild-type TK, the activated TK^Y170E^ phosphomimic and the constitutively inactive TK^K36L^ phosphorylate Tcap comparably and stimulated by Ca^2+^/calmodulin. (i) SDS-PAGE and (ii) autoradiogram of catalysis by samples after Ni^2+^-NTA are shown. (*b*) Phosphorylation assay of TK sterically inhibited by immuno-complexation with an antibody raised against the P+1 loop vicinal to the active site. An antibody (anti-MuRF1) that does not complex TK is included for comparison. (*c*) Untransfected Sf21 cell extracts supplemented with Tcap (but not Ca^2+^/calmodulin) display phosphorylating activity (the stimulation of catalysis upon addition of calmodulin was approx. 25%, this modest activation is likely due to the presence of endogenous calmodulin in the extract). (i) SDS-PAGE and (ii) autoradiogram revealing Tcap phosphorylation. (*d*) (i) Chromatogram and (ii) corresponding SDS-PAGE of Sf21 cell crude extract containing recombinant TK^K36L^ eluted from a Ni^2+^-NTA column. Segregation of phosphorylating activity (cyan) and TK (red) during purification is observed. Bound proteins were eluted with a linear gradient of imidazole (100% buffer B = 0.3 M imidazole; green line) and monitored by A_280_; the resultant chromatogram is in blue. The content of TK^K36L^ in eluted fractions was determined by spot-blot immunoassay using anti-TK P+1 loop antibody. The amount of coloured product quantified densitometrically was proportional to the amount of TK^K36L^ in each fraction (red). Phosphorylation of a Tcap-derived peptide substrate in the presence of calmodulin was quantified in each fraction densitometrically by our standard phosphorylation assay that used [γ-^33^P]ATP and spotting on P81 paper (cyan). The data show that Tcap phosphorylation segregated from TK^K36L^.

In activity assays that used ATP[γ-^33^P], all three TK variants—including the inactive TK^K36L^—showed similar phospho-transfer activities on Tcap and were modestly stimulated by Ca^2+^/calmodulin (figure 1*a*). As an independent validation, we studied the activity of immuno-complexed wild-type TK where its active site had been blocked by a specific antibody directed against the P+1 loop (the efficient complexation of TK by this antibody is shown in the electronic supplementary material, figure S3). Immuno-complexed TK, non-complexed TK samples and non-treated TK controls showed similar levels of activity (figure 1*b*). Hence, activity data from either mutated or immuno-complexed samples together suggested that insect cell preparations catalysed Tcap phosphorylation in a TK-independent way, with kinase activity arising from other component(s) in the cell milieu.

Further evidence that the observed catalysis resulted from a contaminant kinase was derived from TK purification. Recombinant TK segregated from the Tcap phosphorylating activity during fractionation (figure 1*d*) and phospho-transfer activity on Tcap decreased progressively as TK purity increased. The highly pure TK samples showed approximately 80-fold less activity than initial preparations (electronic supplementary material, figure S4). The existence of such contaminating kinase activity was finally confirmed by assaying untransfected Sf21 cell extracts, which displayed notable phosphorylation of Tcap (figure 1*c*). Efforts to identify the contaminant kinase during this work were unsuccessful. A protein that co-purified with TK was speculatively considered as a candidate (electronic supplementary material, figure S4). However, Sf21 cells originate from *Spodoptera frugiperda*, whose genome is not sequenced, and this impeded the identification of the co-purifying protein through proteomic methods. Regardless, these data show that Tcap is a substrate of one or more endogenous kinases in Sf21 cells.

### 3.2. TK produced in *Escherichia coli* is catalytically inactive

**Figure 2.**
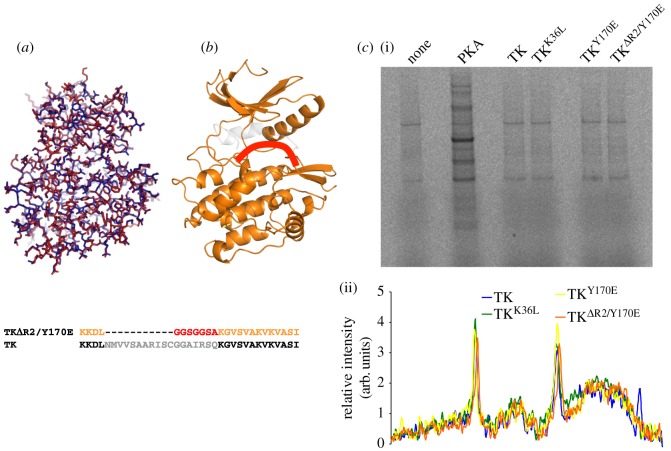
Structural and functional characterization of TK produced in *E. coli*. (*a*) Superposition of the crystal structures of TK expressed in bacteria (red) and insect cells (PDB entry 1TKI) (blue). The overall RMSD for Cα atoms is 0.29 Å. (*b*) Representation of the activated variant TK^ΔR2/Y170E^, where the deleted fraction is in grey and the added loop is shown schematically in red. The sequence exchanges in this variant are shown below. (*c*) Identification of potential TK substrates in differentiating C2C12 cell extracts depleted of endogenous kinases by treatment with FSBA. Protein kinase A (PKA) was used as positive control. (i) Autoradiogram and (ii) densitogram of phosphor-image are provided. The data show no significant differences in labelling pattern when comparing cell extract alone or supplemented with activated forms of TK.

As kinases present in insect cells’ preparations masked the potential catalysis of TK on Tcap, we established the over-production of TK in *E. coli*. To validate that the bacterial form of this sample was viable, we performed a mass spectrometry analysis of TK samples from *E. coli* and Sf21 cells. The data confirmed that neither of the proteins was truncated or otherwise chemically compromised (e.g. mass differences from full mass theoretical values of TK^Y170E^ from Sf21 and *E. coli* cells were +0.7 and +1.8 Da, respectively). The bacterially expressed TK had no phospho-transfer activity on Tcap, nor on the universal kinase substrates myelin basic protein and casein. This agrees with previous observations of inactivity of bacterial TK [16]. To test whether the lack of catalysis resulted from fold defects in the bacterial sample, we elucidated its crystal structure to 2.06 Å resolution (table 1). Crystallization used previous protocols for Sf9-expressed TK [22] and crystals reproduced the lattice parameters of the latter [13]. Following bias removal by simulated annealing, the resulting model of bacterially produced TK was in complete agreement with that of Sf9-expressed samples (RMSD = 0.29 Å for all Cα atoms, calculated with MUSTANG [23]; figure 2*a*). These data confirmed that there are no notable molecular differences between bacterial and eukaryotic forms of TK, and that the absence of catalysis on Tcap signifies that Tcap is not a substrate of TK.

**Table 1.** Data collection and refinement statistics.

	TK
data collection	
space group	P2_1_2_1_2_1_^a^
cell dimensions	
*a*, *b*, *c* (Å)	78.86, 89.73, 113.88^a^
resolution (Å)	28.9–2.06 (2.1–2.06)
no. reflections	49 667 (2681)
*R*_sym_(*I*) (%)	9.1 (56.2)
*I*/*σI*	13.9 (3.6)
completeness (%)	97.9 (95.4)
redundancy	5.7 (5.3)
refinement	
resolution (Å)	28.98–2.06
*R*_work_/*R*_free_ (%)^b^	16.63/20.37
no. atoms	
protein	5235
ligand/ion	28
water	469
*B*-factors (A^2^)	
protein	22.8
ligand/ion	48.4
water	30.7
RMS deviations	
bond lengths (Å)	0.007
bond angles (°)	1.000

^a^For comparison, crystals of eukaryotically expressed TK belonged to space group P2_1_2_1_2_1_ with cell dimensions 78.61, 89.77, 113.32 (a,b,c; Å) and diffracted to 2.0 Å resolution [13,22]. That lattice and the one of bacterially expressed TK in this study are identical.

^b^*R*_free_ set consisted of 1488 reflections, equivalent to 3% of the total number.

We then examined whether muscle cell extracts contained substrates for the highly purified, bacterially expressed TK preparations by performing phosphorylation assays using wt-TK and TK^Y170E^ on extracts from differentiating murine C2C12 myocytes (day 2), as well as gastrocnemius muscle from adult mice (the extracts were depleted of endogenous kinase activities using FSBA). This work did not reveal candidate TK substrates (figure 2*c*). To test whether this result reflected TK auto-inhibition (not expected for TK^Y170E^), we designed the variant TK^ΔR2/Y170E^, where helix R2 was substituted by a flexible loop so that the inhibition of the ATP-binding pocket had been removed (figure 2*b*; nomenclature as in the electronic supplementary material, figure S1). As before, assaying this dually activated variant did not reveal any candidate substrates in cell extracts (figure 2*c*). These results suggest that inactivity is a genuine characteristic of TK.

### 3.3. TK contains atypical residues in its catalytic motifs

To explore the molecular basis of the apparent inactivity of TK, we examined the sequence composition of its active site. A multiple sequence alignment of TK sequences **(**electronic supplementary material, figure S5**)** revealed consistent deviations from canonical active motifs: namely, the bulky hydrophobic residue methionine in position 2 of the V*A*IK motif and a glutamate in the DFG motif. The canonical VAIK motif (reviewed in [24]) is located in strand-β3 and contains the catalytic lysine that ion-pairs the non-transferable α- and β-phosphates of ATP. There is a strong selection for small hydrophobic residues, such as alanine or valine, in position 2 of the VAIK sequence. This residue forms the bottom of the cavity that accommodates the purine heterocycle moiety of ATP. A bulky residue at this position, as methionine in TK, might sterically hinder ATP binding or lead to a non-catalytic binding mode. As TK has been reported to bind ATP/Mg^2+^ [16], we explored whether the complex might be unproductive by modelling the catalytic domain of TK (in the absence of the CRD) in its predicted active conformation. In the latter, the N-terminal lobe has closed onto the bound ATP/Mg^2+^ substrate as typically observed in catalytic kinases (electronic supplementary material, §S6). To validate the modelling protocol, the invertebrate homologue twitchin kinase (TwcK), which has well-established catalysis [25–27] and conventional active site features, was used here as reference. In modelling, we used as template the active conformation of the ATP/Mg^2+^-bound death-associated protein kinase, which is closely related to titin-like kinases [28]. The model of active TwcK so produced suggests that it follows canonical patterns, where lobe closure leads to the formation of a regular ATP-binding cavity. By contrast, the closed model of TK shows an unusual ATP cavity, where shape fits poorly the ATP ligand and M34 occludes the adenine-binding pocket (electronic supplementary material, figure S6). We conclude that in TK, M34 hinders the adoption of a canonical ATP-bound, closed-lobe conformation as characteristic of active kinases.

**Figure 3.**
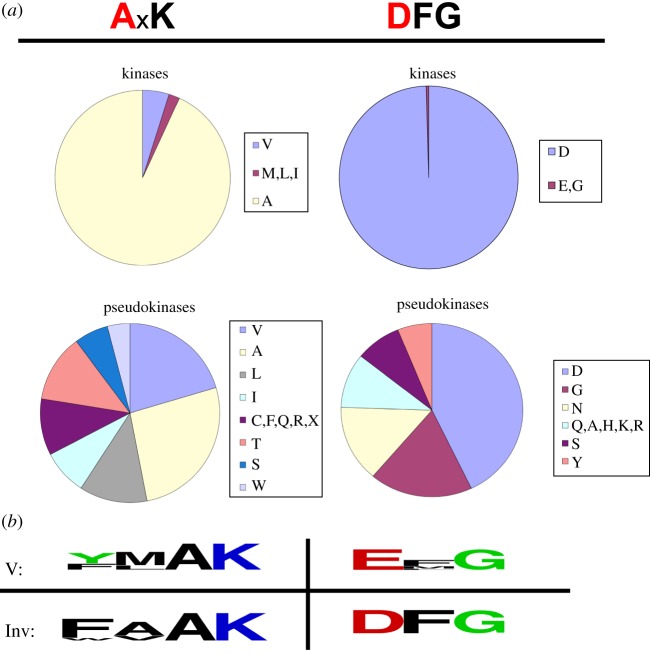
TK contains atypical residues in catalytic motifs. (*a*) Distribution of residues in position 2 of the V*A*IK motif and position 1 of the *D*FG motif in protein kinases of the human kinome. The classification of kinases and pseudokinases was taken from [32]. TK, the only human kinase containing an EFG motif, is misclassified as active kinase due to previous reports of catalysis [10,13,16]. Other kinases with deviant catalytic motifs are: CASK (*G*FG motif); ATR (x*M*xK motif); LMR2, NEK8 and RIPK1 (IxK); and DNAPK, FRAP and SMG1 (LxK). (*b*) Commonly occurring residues in the VAIK and DFG motifs of titin kinases from vertebrate (V) and invertebrate titin-like kinases (Inv). (Sequences for representative kinases of each group are given in the electronic supplementary material, figure S5.)

In the DFG motif, aspartate chelates the magnesium ion that commonly coordinates the β-phosphate of ATP. This residue is conserved across most members of the protein kinase-like superfamily [29]. Although glutamate and aspartate are chemically similar, this substitution is sufficient to inactivate phospho-transfer in kinases [30,31]. In the human kinome [32], the deviant residues found in TK are extremely rare among active kinases (figure 3). TK is the only known human kinase that contains glutamate instead of aspartate in the DFG motif, while ATR kinase is the only other human kinase containing a methionine in position 2 of the VAIK motif (but its level of activity is unclear). By contrast, among pseudokinases there is no marked selection for given residues in position 2 of the VAIK motif or aspartate in the DFG signature. Thus, the YMAK/EFG signatures clearly point to irregular catalysis in TK.

Interestingly, an exploration of sequences of TK-like kinases from invertebrates that included twitchin and TTN-1 kinases from nematodes and molluscs, and projectin from insects, showed that these homologues contain canonical catalytic motifs characteristic of active kinases (electronic supplementary material, figure S5). As mentioned, the TwcKs from *Aplysia* and *Caenorhabditis elegans* exhibit high levels of catalysis [25–27]. This suggests an evolutionary dichotomy where invertebrate members of the TK family are functional enzymes, but the vertebrate counterparts are catalytically compromised pseudokinases.

### 3.4. Transfer of MAK/EFG TK signature motifs to twitchin kinase abolishes catalysis

**Figure 4.**
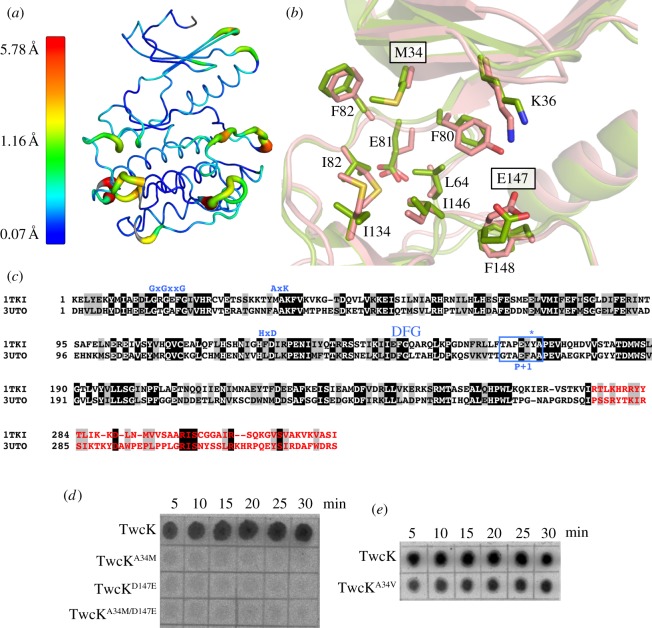
Comparison of TK and TwcK active sites. (*a*) Structural superposition of TK and *ce*TwcK (PDB entry 3UTO). Ribbon thickness and colouring indicate the RMSD values of the superposition as given in the accompanying scale (minimum, maximum and average values are shown). The structural agreement is excellent overall, including active site regions; divergences only occur in peripheral loop areas. (*b*) Detailed comparison of the ATP-binding pockets of TK (green) and *ce*TwcK (pink) (numbering corresponds to TK). Boxed labels indicate TK residues that were trans-engineered into *ce*TwcK. (*c*) Structure-based sequence alignment of the catalytic domains of human TK and *ce*TwcK corresponding to the superposition displayed in (*a*,*b*). Identical residues are highlighted in black and closest conservation is in grey. The canonical composition of functional motifs is shown in blue, the P+1 loop is boxed and the tyrosine residue undergoing phosphorylation in TK is indicated with an asterisk. The CRD is in red. (*d*) Comparative autoradiogram of catalysis by *ec*Twck and its variants TwcK^A34M^, TwcK^D147E^ and TwcK^A34M/D147E^ carrying TK residues in their ATP-binding pockets. The time course shows phosphorylation of a MLC-derived peptide. (*e*) Comparative autoradiogram of the catalysis from *ec*Twck and TwcK^A34V^. The latter carries the non-inactivating valine residue commonly found in the ATP-binding pocket of TwcK from molluscs.

To study the role of the atypical, conserved methionine and glutamate residues in the active site of TK, we substituted these for their canonical equivalents in the variants TK^E147D^, TK^E147D/M34A/R129K^ and TK^E147D/M34A/R129K/Y170E^ (R129 is normally a conserved lysine residue in the catalytic loop of active kinases of the titin-like family; we speculated that in TK, the exchange to arginine might be coupled to other divergences in the catalytic motifs of this kinase). Of these variants, only TK^E147D^ could be produced soluble in *E. coli*. However, it was not active when assayed on Tcap or the generic kinase substrates myelin basic protein and casein. Therefore, we performed the reverse experiment, mutating the TK residues into *ce*TwcK. The latter is a close homologue of human TK that is well characterized structurally and biochemically [25–27]. *ce*TwcK (bacterially expressed) exhibits high levels of catalysis when assayed on a model peptide substrate derived from myosin light chain (MLC) protein. The catalytic domains of *ce*TwcK and TK share 40% sequence identity and 65% conservation (figure 4*c*), and, accordingly, high structural similarity (RMSD for Cα atoms = 1.29 Å when comparing the structure of TK in this work and PDB entry 3UTO using MUSTANG [23]; figure 4*a*). The active sites of these two kinases are in close structural agreement, particularly their ATP-binding pockets, their β3 strands and the ^D^/_E_FG motifs (figure 4*b*). Thus, we concluded that *ce*TwcK is a suitable template to investigate the effect of the unusual motifs of TK on catalysis.

The mutations A34M and/or D147E were introduced in *ce*TwcK (variants *ce*TwcK^A34M^, *ce*TwcK^D147E^ and *ce*TwcK^A34M/D147E^) and phosphorylation assays carried out on the MLC-derived peptide substrate. The double mutant *ce*TwcK^A34M/D147E^ was generated to account for the possible compensatory coevolution of these substitutions in TK. In contrast to wild-type *ce*TwcK, which showed its characteristically high activity, all generated mutants lacked measurable phospho-transfer (figure 4*d*). To confirm that this result was not due to unsuspected distortions of the active site of *ce*TwcK caused by the mutations, we generated the variant *ce*TwcK^A34V^ as positive control. In this construct, the alanine in the VAIK motif is replaced by a valine residue, which is found in the catalytically active TwcKs from molluscs (electronic supplementary material, figure S5) as well as 5% of the active members of the human kinome [32]. As expected, *ce*TwcK^A34V^ retained notable levels of catalysis (figure 4*e*). The inactivation induced by M34 agrees with studies on B-Raf where substitution of the corresponding residue with a bulky phenylalanine group abolished ATP binding [33]. These results indicate that the two atypical active site residues M34 and E147 in TK are sufficient to inactivate it.

### 3.5. TK supports the interaction of M-line titin with the E3 ubiquitin ligase MuRF1

**Figure 5.**
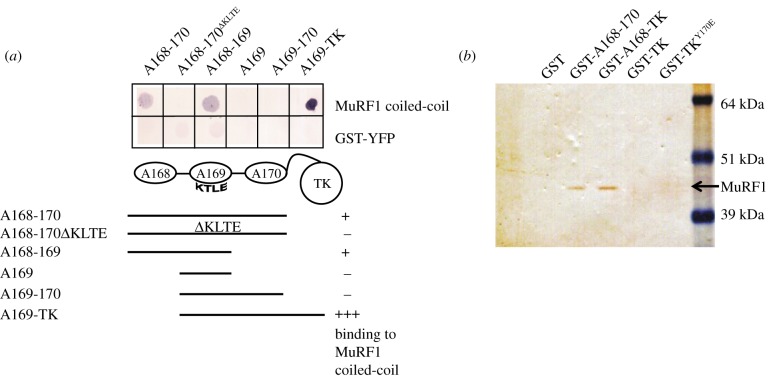
Dissection of TK/MuRF1 molecular interactions. (*a*) Identification of the MuRF1–titin interaction region by filter-binding assay. The helical domain of MuRF1 interacts with its established docking site A168–A170 and the C-terminally truncated version A168–A169, but not with the loop mutant A168–A170^ΔKTLE^, the N-terminally truncated A169–A170 or the single domain A169. Binding to A169–A170 is restored and enhanced when TK is included in the construct (A169-TK). The latter is used here in 10-fold lower quantity than the other samples. (*b*) Pull-down assay using skeletal muscle extract demonstrates that A168–A170 and A168-TK bind endogenous MuRF1 efficiently, but that the binding is stronger in the presence of TK. Neither GST nor TK alone are capable of pulling down endogenous MuRF1.

Based on these findings, we reassessed how TK might contribute to muscle signalling in non-catalytic ways. The binding of the muscle-specific E3 ubiquitin ligase MuRF1 in the vicinity of TK is well documented [7,8]. MuRF1 binds a tandem of Ig-Ig-Fn domains, A168–A170, preceding the TK domain. The binding is mediated by the C-terminal helical domain of MuRF1 and determined by the presence of a loop with sequence KTLE in the titin domain A169 [8]. Here, we generated titin constructs comprising variations of the established MuRF1 docking site as well as TK (figure 5). A168–A170 and its C-terminally truncated variant A168A–A169 interacted with MuRF1, while the loop mutant A168–A170^ΔKTLE^ (where the KTLE motif had been mutated to AAAA), N-terminally truncated A169–A170 and the single A169 domain did not display detectable interaction. Interestingly, when the fragment A169–A170 (which does not bind MuRF1 detectably) was extended to include TK, MuRF1 binding was restored and stronger than observed for the established A168–A170 locus (figure 5*a*). It is worth noting that the concentration of A169-TK was 10 times lower than that of the other constructs in the assay, indicating that TK markedly boost the MuRF1 interaction.

We further confirmed these findings via pull-down assays on skeletal muscle extracts (figure 5*b*). Here, A168–A170 and A168-TK were able to pull down the endogenous MuRF1 but, as before, the sample containing TK showed a stronger binding. TK alone (either as wild-type or Y170E phosphomimic) did not interact with MuRF1 detectably. Taken together, the data indicate that the MuRF1 docking site in M-line titin spans the region A168-TK, which constitutes an extended multi-domain scaffold of approximately 18–20 nm length. Our data suggest that this scaffold contains two binding loci: a high-affinity site spanning A169-TK and the established A168–A169 site that has lower apparent affinity. It is likely that both sites are involved simultaneously in the recruitment of a same MuRF1 molecule, as the length of the A168-TK region agrees well with the rod-like dimensions of MuRF1 revealed by EM (approx. 17 ± 3 nm length) [34]. Taken together, the data suggest that TK might play a dominant role in the recruitment and retention of MuRF1 in the sarcomeric M-line.

## 4. Discussion

The signalling context of TK in the sarcomere has remained elusive since its discovery over two decades ago [35]. Efforts to identify activators and substrates of this kinase have only yielded candidates of doubtful physiological relevance. Tcap [13], nbr1 and p62 [10] have been proposed to be phosphorylation substrates of TK. Of these, only Tcap is phosphorylated to a significant degree. However, in the sarcomere Tcap is commonly found as part of a larger protein complex in the periphery of the Z-disk, approximately 1 µm away from TK in the M-line. The non-diffusible nature of both enzyme and substrate has cast doubt on the *in vivo* significance of the interaction. Here, we provide evidence that TK is an inactive pseudokinase. Our data derive from the testing of wild-type TK, active phosphomimics (TK^Y170E^ and TK^ΔR2/Y170E^) and inactivated variants (TK^K36L^) expressed in Sf21 eukaryotic cells and in the *E. coli* bacterial system. These TK forms were assayed for activity on Tcap as well as on inactivated extracts from developing myocytes and mature muscles. This did not reveal catalysis that could be attributed to TK. Structurally, two atypical residues (M34 and E147) that are conserved in vertebrate TKs proved to be individually capable of disabling the highly active TK homologue, TwcK. These data, together with our identification of a contaminant kinase activity in the insect cell preparations commonly employed for the production of TK, lead us to deduce that the previous assignments of catalysis and substrates to TK is questionable. Notably, the contaminant kinase is activated by calmodulin (figure 1), a characteristic previously attributed to TK [10,13]. Interestingly, a recent report shows that Tcap is a substrate of protein kinase D and CAMK-II *in vitro* [36]. That work identifies Ser157 as one of the phosphorylation sites of those kinases in Tcap. The phosphorylation of Ser157 has also been attributed to TK [18]. This brings strong support to our view that previous experimentation on TK has been troubled by the presence of contaminant kinase(s) in the sample preparations.

The obstruction of functional studies by contaminant kinases in recombinant preparations from insect cells is not rare. For example, Hamel *et al*. [37] found that a contaminant kinase in preparations of a substrate masked the activity of MAPK, invalidating phosphorylation assays of the latter. A further case is that of G-protein preparations, which are commonly contaminated with a lipid kinase [38]. Scientifically misinterpreted, the contaminant activity was initially attributed to integrin-linked kinase (ILK), which later was shown to be an inactive pseudokinase [39]. The study of potential catalysis in pseudokinases through loss of function variants can also yield misleading results. For example, conventional KtoA active site mutants of BUBR1 affected the mitotic checkpoint [40,41], which was later shown to result from impaired conformational stability and not catalytic inactivation [42]. In ILK, the KtoA mutation led to renal agenesis during kidney development [43], even though ILK has no catalytic activity. It was then shown that pathogenic mutations in ILK act by impairing the structural integrity of this kinase [39]. Similarly, transfection of wild-type TK in BHK-Bi cells stimulated transcription of myomesin, BNP and c-fos reporter genes, but this effect was abolished in the variant TK^D127A^ missing the putative catalytic aspartate [10]. Our data suggest that these cellular effects might be caused by the alteration of TK's scaffolding function and not its catalysis. Less clear is the source of the apparent loss of catalysis in *in vitro* preparations of TK^K36A^ previously reported [13]. We tentatively speculate that possible changes in fold stability (electronic supplementary material, §S2) or expression yields in that variant with respect to TK^K36L^ used in this study might have influenced the retention of the co-purifying contaminant kinase during chromatography.

Our findings prompt a reassessment of how TK as an inactive pseudokinase contributes to muscle stress signalling as its hypothesized mechanoactivation mechanism is unlikely to relate to catalytic activity. Pseudokinases are known to participate in cellular pathways as regulated scaffolds for the assembly of signalling complexes [44]. In TK, mechanical deformations might regulate its interaction with associated proteins. This appears potentially applicable to its binding of MuRF1, where multi-domain contact points make the interaction sensitive to the stretch-induced conformation of the titin chain. Our data suggest that TK and MuRF1 are part of a shared stress-signalling pathway. Integrating previous knowledge of MuRF2 recruitment to the TK signallosome [10], it now appears likely that both MuRF1 and MuRF2 can occupy the TK scaffold. This might result in TK serving as a switch to gate MuRF1/MuRF2 signalling, where speculatively mechanical arrest may release MuRF2 and recruit MuRF1. MuRF1 is transcriptionally upregulated by multiple myopathic stimuli in addition to mechanical inactivity. By serving as a cross-talk node, TK might align the general myopathic response of MuRF1 with the mechanical response of MuRF2, possibly initiating similar cellular processes in response to otherwise divergent stimuli.

**Figure 6.**
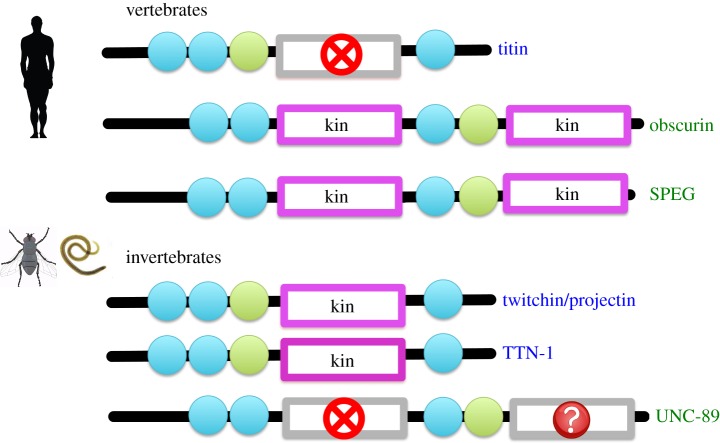
Distribution of active kinases and inactive pseudokinases in titin-like filaments from vertebrate and invertebrate muscle. For each filament, the following domains are shown: Ig (blue circles), Fn3 (green circles), active kinases (magenta boxes) and inactive pseudokinases (grey boxes). Pseudokinases where inactivity is tentatively suggested but cannot be as reliably predicted are indicated with a question mark.

Finally, the inactivity of vertebrate TK contrasts markedly with the high levels of *in vitro* catalysis of its invertebrate homologues, TwcK, TTN-1 and projectin. Interestingly, this difference correlates with a similar contrast in obscurin kinases, also members of the TK kinase family. Obscurin (and the invertebrate homologue UNC-89) contains two kinases, PK1 and PK2. In vertebrates, PK1 and PK2 have canonical active sites and are catalytically active [45,46]. In invertebrates, PK1 is inactive as it lacks catalytic residues and PK2 has a degenerated active site indicating that its activity might be modest [46]. This inverse correlation in titin-like and obscurin-like kinases in vertebrate and invertebrate organisms (figure 6) suggests that stretch-activated phospho-transfer might reside in different filament systems in muscles across animal phyla. Future studies are required to understand the patterns of activity and scaffolding in these kinases in terms of muscle responses to stress stimuli.

## 5. Material and methods

### 5.1. Cloning

TK (residues 32 172–32 492; UniProtKB Q8WZ42) and its variants were cloned into the pET-Trx1a vector (EMBL collection) using NcoI and Acc65I restriction sites. This vector fuses a His_6_-tagged thioredoxin and a tobacco etch virus (TEV) protease cleavage site N-terminally to the target construct. For expression in Sf21 cells, the full-length protein encoded by the pET-Trx1a vector, including the N-terminal fusion, was cloned into pFastBac ET vector (EMBL). The NcoI compatible PscI site was introduced at the 5′-end of the construct and cloned into a linearized vector digested with NcoI/Acc65I restriction enzymes. The variant TK^ΔR2/Y170E^ was constructed by replacing residues 291–309 forming the regulatory α-helix R2 with the sequence GGSGGSA using TK^Y170E^ as a template. To ease structural annotation, residue 32 172 is taken here as residue 1.

TwcK (residues 6251–6537; UniProtKB Q23551) and its variants as well as full-length human Tcap (UniProtKB O15273) were cloned into the pETM-11 vector that adds a His_6_-tag and a TEV protease cleavage site prior to the inserted protein. For compatibility with TK, residue 6251 in TwcK is considered here as 1 (based on the structural alignment in figure 4*c***)**. GST-tagged titin A168–170 (residues 31 854–32 155), A168-TK (residues 31 854–32 492), TK and TK^Y170E^ were cloned into pETM-20 vector (EMBL collection). All constructs were verified by sequencing.

### 5.2. Recombinant protein production

Kinase samples were expressed in *E. coli* SoluBL21 (Genlantis) in TB medium supplemented with 50 μg ml^−1^ kanamycin. Cultures were grown at 37°C to an OD_600_ = 1. Upon cooling to 16°C, expression was induced with 0.2 mM IPTG and growth continued for a further 18 h. Cells were harvested by centrifugation. The pellet was resuspended in lysis buffer (20 mM HEPES pH 8, 250 mM NaCl, 5 mM imidazole, 0.2% NP40, 2 mM β-ME, 2 mM PMSF), supplemented with DNAse I, lysozyme and 1 mM PMSF, and lysed by sonification. The supernatant was applied to a Ni^2+^-NTA column (Qiagen) pre-equilibrated in lysis buffer and proteins eluted with 300 mM imidazole in lysis buffer without detergent. Tag removal was by incubation with TEV protease in 25 mM Tris pH 8, 50 mM NaCl, 5 mM DTT, overnight at RT. Proteins were further purified using ion exchange chromatography (HiTrap SP for TK; HiTrap Q for TwcK) and gel filtration on a Sephadex S75 16/60 column (GE Healthcare). Samples were stored at 4°C until further use.

Tcap was produced as TwcK but without tag removal or gel filtration. GST-tagged proteins were purified using Glutathione-Sepharose (GE Lifesciences) resin.

For eukaryotic TK expression (Protein Expression and Purification Facility of EMBL), Sf21 cells were transfected with the bacmid DNA construct of TK or its variants according to manufacturer instructions (Invitrogen Bac-to-Bac system). Transfected cells were incubated for 3 days at 27°C and the supernatant, containing recombinant virus, was collected. Amplified virus (10 ml) was added to 1 × 10^6^ Sf21 cells and incubated at 27°C for 3 days. Sf21 cells were harvested 72 h post-infection and stored at −20^o^C until protein purification, which followed the procedure above.

### 5.3. Crystal structure determination

As before [13,22], crystals were grown at 21°C by the hanging drop method in 24-well VDX plates (Hampton Research). Drops contained 1 µl protein solution (12 mg ml^−1^) and 1 µl reservoir consisting of 1.6 M Na/K tartrate, 25 mM sodium acetate pH 4.9, 25 mM imidazole pH 7.5, 2.5% PEG 400. Crystal optimization used seeding into solutions with 1.2 M Na/K tartrate but of otherwise identical composition. For data collection, crystals were flash frozen in liquid nitrogen using mother liquor supplemented with 30% (v/v) glycerol. X-ray diffraction data were collected at 100 K on beamline I04, DIAMOND (Didcot), at *λ* = 0.9763 Å on an ADSC Quantum detector. The data were processed with XDS/XSCALE [47] (table 1). As before, crystals contained two molecular copies in the asymmetric unit (RMSD = 0.14 Å, in MUSTANG [23]). Phasing was by molecular replacement in Phaser [48] using one copy of eukaryotic TK (PDB entry 1TKI) as search model. Model refinement and solvent building was in PHENIX [49], applying simulated annealing with a starting temperature of 5000°C. Manual building was in COOT [50].

### 5.4. *In vitro* phosphorylation assays

Phosphorylation assays were performed in 20 µl assay buffer (20 mM Tris–HCl pH 7.4, 10 mM magnesium acetate, 0.05% NP40, 0.1 mM DTT, 0.2 mg ml^−1^ acetylated BSA) containing 0.4 mM ATP (0.2 µCi/reaction of [γ-^33^P]ATP) at 30°C. Tcap, MLC-derived peptide (KKRARAATSNVFS), Tcap-derived peptide (RRSLSRSMSQEAQRG), casein and myelin basic protein were tested at 4 µg/reaction. Where indicated, the reaction mixture was supplemented with 0.5 mM CaCl_2_ and 0.4 µg/reaction calmodulin from bovine testis (Sigma-Aldrich). For reaction mixtures that assayed peptide substrates, an aliquot was withdrawn at indicated time points and spotted on EDTA impregnated P81 phosphocellulose paper (Whatman). The latter was washed extensively with 75 mM orthophosphoric acid and once with ethanol, dried and exposed to a phosphoscreen. Reaction mixtures with protein substrates were first separated on SDS-PAGE, the gel briefly stained, dried and exposed to the phosphoimager screen. Screens were imaged with a Fujifilm BAS 2500 phosphoimager and images processed using AIDA (Raytest).

### 5.5. Preparation of inactivated C2C12 extracts

C2C12 cells were grown on gelatin-coated plastic flasks in DMEM, 10% fetal calf serum, 1× ITS, penicillin and streptomycin. For differentiation, the medium was changed to DMEM, 2% horse serum, penicillin, streptomycin once the cells reached 70–90% confluence. For FSBA extract inactivation, cells were lysed in 50 mM Tris pH 7.4, 150 mM NaCl and 1% NP-40. Clarified lysate was depleted of endogenous ATP on a desalting column (GE Lifesciences) and treated with 20 mM FSBA at 30°C for 1 h. FSBA was removed by buffer exchange into phosphorylation assay buffer (above) supplemented with 5 mM DTT.

### 5.6. Pull-downs using recombinant titin fragments on muscle extracts

Quadriceps tissue from adult mice was pulverized under liquid nitrogen and the powder homogenized in 20 mM Tris pH7.5, 100 mM NaCl, 2 mM β-ME and complete protease inhibitor (Roche). Extraction proceeded for 90 min on ice. The resulting extracts were clarified at 3000*g* for 30 min, aliquoted, frozen in liquid nitrogen and stored at −80°C.

For GST pull-downs, 50 µg each of GST, GST-A168–170, GST-A168-TK, GST-TK and GST-TK^Y170E^ were immobilized on 30 µl of Glutathione-Separose beads and washed with PBS, 0.1% NP40. Extracts were thawed on ice and spun for 1 min at 21 000*g*. The supernatant was diluted by adding three volumes of PBS, 0.1% NP40. Next, 1 ml of diluted extract was mixed with 30 µl of beads loaded with a given titin fragment and incubated for 16 h at 4°C under light stirring. Mixtures were spun for 1 min at 1000*g*, supernatants were removed and the beads washed 3× with 1 ml PBS, 0.1% NP40. After a final wash with PBS, supernatants were removed and the beads resuspended in SDS sample buffer. Bound material was examined by SDS-PAGE and Western blotting using a MuRF1-specific antibody.

### 5.7. Filter-binding assay

Purified His_6_-MuRF1-coiled-coil (aa169–263, Q969Q1; reported in [8]) was biotinylated with NHS-PEG_4_-Biotin (Thermo Scientific). To remove unreacted biotin, the sample was filtered twice through a PD-10 column (GE Healthcare) in PBS. One millimolar DTT and 50% glycerol were added and the sample frozen until further use.

Purified titin constructs were spotted (approx. 1 µg) on nitrocellulose membranes pre-wetted with TBS. The filter was blocked by TBST/5% milk, washed with TBST and incubated for 3 h with biotinylated MuRF1-coiled-coil at a concentration of 0.5 µg ml^−1^ in TBST/1% milk. The membrane was washed in TBST and incubated for 1 h in TBST containing 2 µg ml^−1^ streptavidin conjugated to alkaline phosphatase (Thermo Scientific). After washes in TBST and a final wash in TBS, the detection with NBT/BCIP was done as described by the supplier (Roche).

## Supplementary Material

Supplementary Material

## Data accessibility

Model coordinates and structure factors have been deposited with the PDB (entry 4JNW).
