# Supplementary material for: Titin kinase is an inactive pseudokinase scaffold that supports MuRF1 recruitment to the sarcomeric M-line
Source: Open Biol. 2014 May 21;4(5):140041. doi: 10.1098/rsob.140041 (PMC4042850; doi:10.1098/rsob.140041)
Supplement: Supplementary Material [file rsob140041supp1.pdf]

Titin kinase is an inactive pseudokinase scaffold that supports MuRF1 recruitment to the sarcomeric M-line

Julijus Bogomolovas[1,2], Alexander Gasch[1], Felix Simkovic[2], Daniel J Rigden[2], Siegfried Labeit[1], Olga Mayans[2*]

Supplementary Materials

Fig S1: Overall fold representation of TK

The catalytic kinase core is shown in blue with an accompanying surface representation. The C-terminal regulatory domain (CRD) is colored yellow and its αR2 helix, that binds deeply into the ATP binding cavity, is indicated with a pointer. The lysine residue central to ATP binding and catalysis (K36), the putative catalytic aspartate residue (D127) and the inhibitory tyrosine from the P+1 loop (Y170) are displayed.

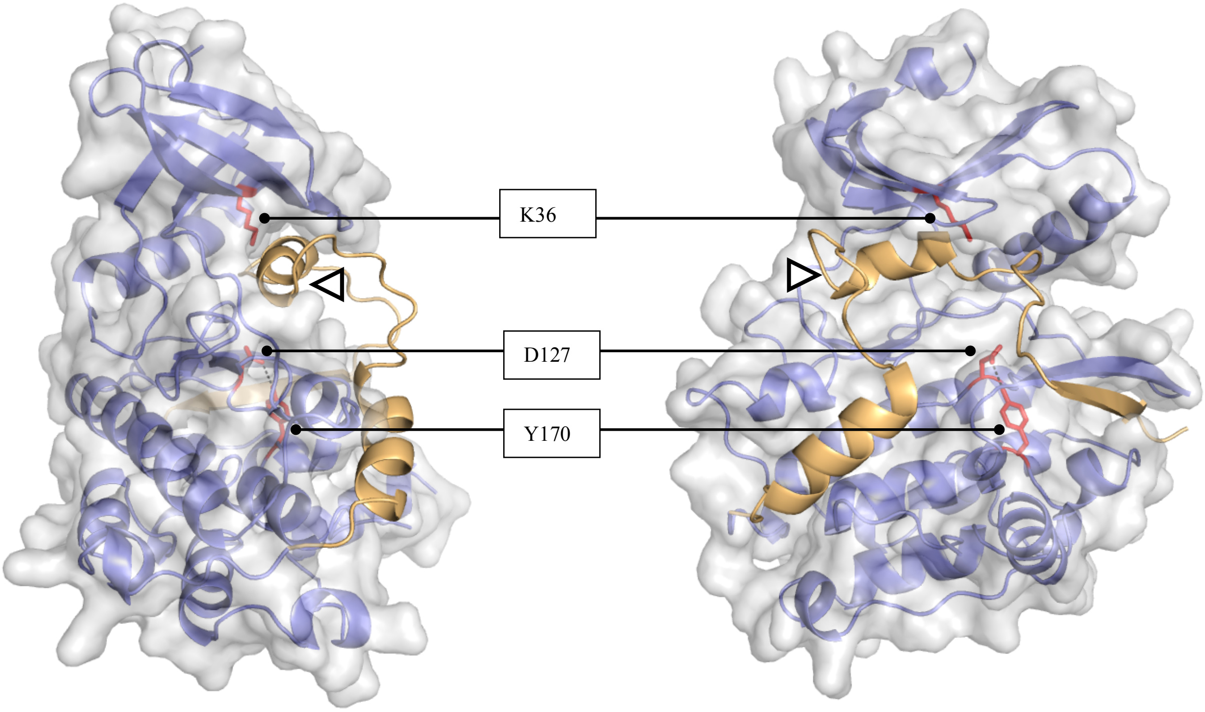

Section S2: Stability study of TK and its variants TK^{K36A} and TK^{K36L}

Methods: Energy changes (ΔΔG) for the replacement of lysine K36 in the ATP-binding pocket with each possible amino acid type were estimated using FoldX (1). Experimental protein stability for selected mutated variants was derived from differences in their melting temperature (Tm) monitored by Differential Scanning Fluorimetry (DFS). DFS measurements were performed using a Mx3005p RT-PCR machine (Stratagene). Purified TK, TK^{K36A} and TK^{K36L} samples were assayed in 25 μl buffer consisting of 10 mM HEPES pH 7.5, 150 mM NaCl in 96-well plates. SYPRO-Orange (Invitrogen) was added at a dilution 1:1000. Fluorescence was monitored

(λex=465 nm, λem=590 nm) from 25 °C to 85 °C at 1 °C/min increment. Each measurement was done in triplicate and Tm values determined using a modified Boltzmann equation with linear correction of baseline drifts (2).

Fig S2: Stability of TK lysine mutants
a. Energy changes (ΔΔG) for the replacement of lysine K36 by every other amino acid calculated using FoldX. The values confirm observations that the conventional replacement of K36 to alanine is poorly tolerated leading to the destabilization of the TK fold. A replacement of this residue by leucine, however, is predicted to agree well with the structure of TK; **b.** DSF denaturation traces recorded at λ_{em}=590 nm. Substitution of K36 for alanine (TK^{K36A}) had a destabilizing effect (ΔTm= 6.6 °C) on TK, whereas the stability of TK^{K36L} (ΔTm= 2.2 °C) was approximately equivalent to that of wild-type TK.

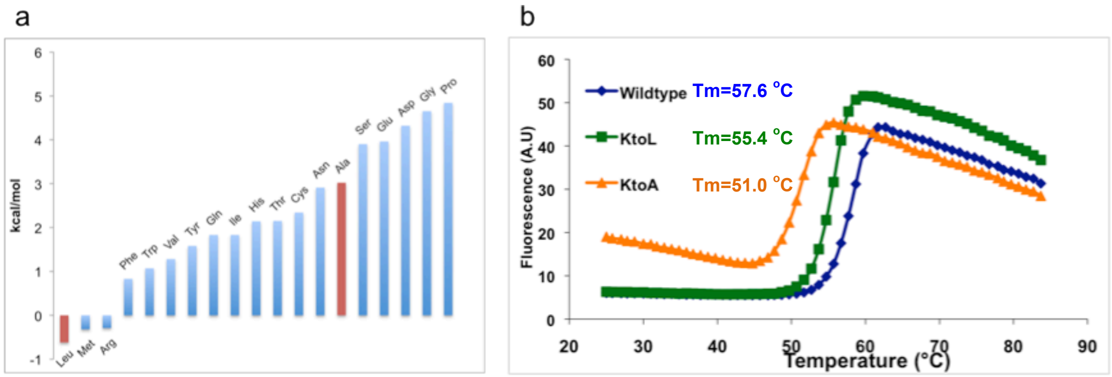

Section S3: Production of anti-TK and anti-MuRF1 antibodies

Production of antibodies: Antigen affinity purified rabbit antibodies against a synthetic peptide corresponding to amino acids 2-15 of MuRF1 or amino acids 163-177 of TK were obtained from BioGenes (Berlin, Germany).

Dot blot assay: TK was expressed in E. coli with an N-terminal hexahistidine-tag, purified on Ni^{2+}-NTA agarose, and then spotted on nitrocellulose membranes pre-wetted with TBS (approximately 1 µg each). Filters were incubated with purified rabbit antibodies, N-terminal MuRF1-peptide (a) or TK (b), followed by incubation with Alkaline Phosphatase coupled anti-rabbit antibodies. Blocking and antibody incubations were in TBST/5% BSA and washes in TBST.

Figure S3: Dot blot assays of anti-TK and anti-MuRF1 antibodies. anti-MuRF1 antibodies (**a**) recognize the spotted MuRF1sample, but not the TK constructs, whereas anti-TK antibodies recognize TK, but not MuRF1 or titin Ig/Fn3 domains (titin sample).

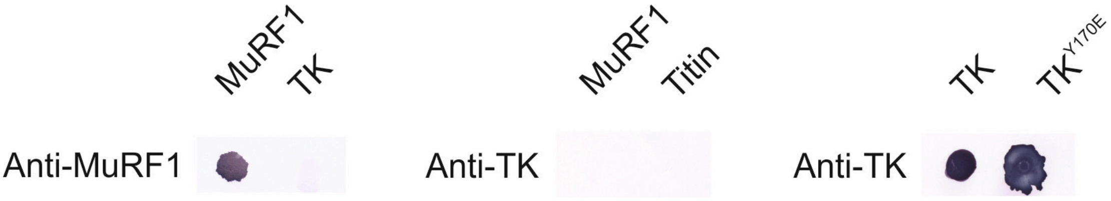

Fig S4: Phospho-transfer activity does not segregate with TK in purification

In vitro Tcap phosphorylation assays using **a.** partially purified TK samples after one-step affinity chromatography and **b.** TK samples thoroughly purified using the three-step chromatography protocol described in Methods. Both a. and b. show: Coomassie stained SDS-PAGE of reaction mixture (left), autoradiogram of phosphorylated samples (center) and SDS-PAGE of the TK sample prior to being added to the reaction mixture (right). A same amount of total protein content (as estimated using A_{280}) was used in both experiments; **c.** Densitogram showing a quantification of Tcap phosphorylation according to autoradiograms obtained in carefully matched experimental set-ups to allow for comparison. Namely, a same amount of total protein (0.5 μg), radioactivity (0.2 μCi) and substrate/additives was used per reaction; equivalent pre-cast commercial gels (NuPAGE®, Invitrogen) were used; gels were always exposed for 6 hrs upon drying following a same protocol and images obtained using the same scanning parameters in the same imaging machine. The quantitation shows that purified TK samples displayed an estimated 80-fold less phosphor-transfer activity than partially pure TK samples. A contaminant (red pointer) co-purifies with TK at low levels. This contaminant has a Mw slightly over 62 kDa (referenced to Mw markers). We speculate that this might be the kinase acting on Tcap, but could not rule out that might be a chaperone or other host protein binding to TK. Efforts to identify this protein through proteomic approaches were not successful as the genome of *Spodoptera frugiperda* is not available.

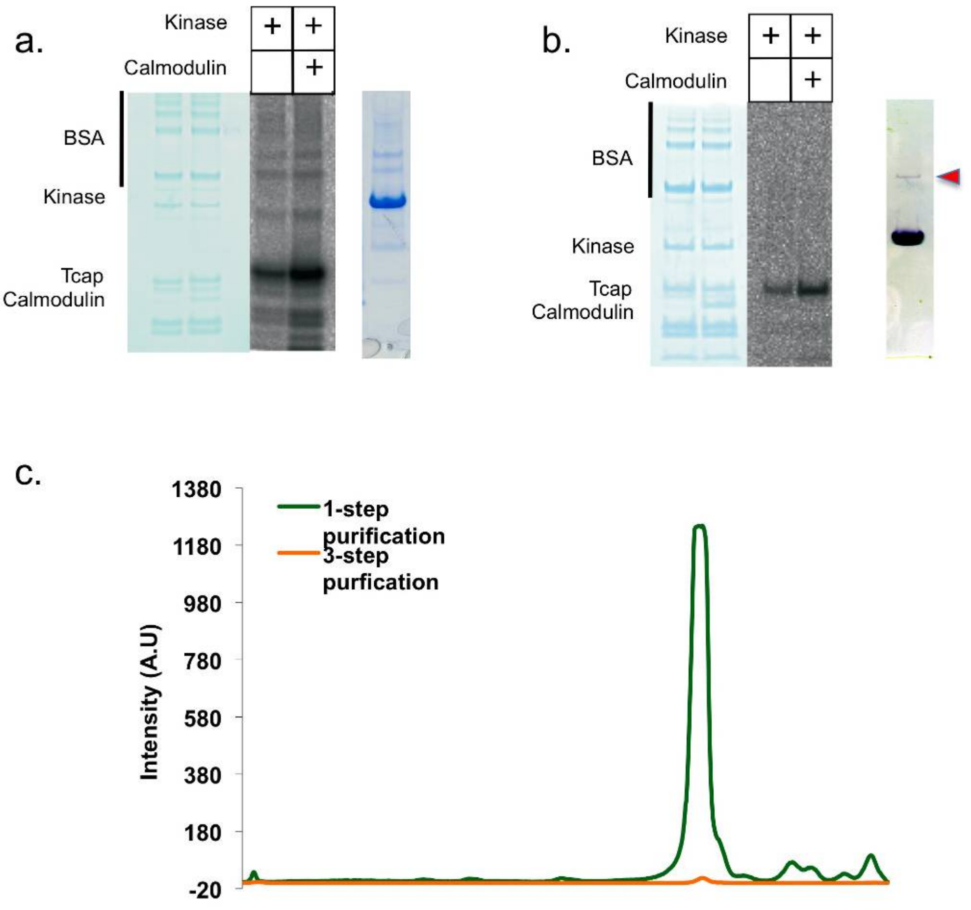

Fig S5: Sequence conservation of VAIK and DFG motifs in TK-like kinases from vertebrates and invertebrates.

Vertebrate titins	VAIK		DFG
Homo_sapiens	CVETSSKKTYMAKFVKVKGTDQVLVK	....	TIKIIEFGQARQL
Ailuropoda_melanoleuca	CVETSSKKTYMAKFVKVKGTDQVLVK	....	TIKIIEFGQARQL
Anolis_carolinensis	CVETATKKTYLAKFVKVKGADQVLVK	....	IIKIIEFGQARQL
Bos_taurus	CVETSSKKTYMAKFVKVKGTDQVLVK	....	TIKIIEFGQARQL
Callithrix_jacchus	CVETSSKKTYMAKFVKVKGTDQVLVK	....	TIKIIEFGQARQL
Canis_familiaris	CVETSSKKTYMAKFVKVKGTDQVLVK	....	IIKIIEFGQARQL
Cavia_porcellus	CVETSSKKTFMAKFVKVKGTDQVLVK	....	VIKIIEFGQARQL
Danio_rerio_A	CIETSSEKTYMAKFVKVKGADQALVK	....	NVKIIELGQSRHL
Danio_rerio_B	SIEISSKKTFLAKFIKVKGADRELVA	....	TIKIIEMGQARLL
Dasypus_novemcinctus	CVETSSKKTYMAKFVKVKGTDQVLVK	....	TIKIIEFGQARQL
Dipodomys_ordii	CVETSSKKTFMAKFVKVKGTDQVLVK	....	TIKIIEFGQARQL
Echinops_telfairi	CVETSSKKTYMAKFVKVKGTDQVLVK	....	TIKIIEFGQARQL
Equus_caballus	CVETSSKKTYMAKFVKVKGTDQVLVK	....	VIKIIEFGQARQL
Erinaceus_europaeus	CVETSSKKTYMAKFVKVKGTDQVLVK	....	IIKIIEFGQARQL
Felis_catus	CVETSSKKTYMAKFVKVKGTDQVLVK	....	SIKIIEFGQARQL
Gadus_morhua_A	CVEIATKKTFMAKFIKVKGMDRELVL	....	ELKIIEMGQARLL
Gadus_morhua_B	CVEKSSERTYMAKFVKVRGADQAIVK	....	NVKIIELGQCRHL
Gallus_gallus	CVEAVSKKTYLAKFVKVKGADQVLVK	....	VVKIVEFGQARQL
Gasterosteus_aculeatus_A	CTEIATKKTFMAKSIKVKGTDRELVL	....	TTKIIEMGQARLL
Gorilla_gorilla	CVETSSKKTYMAKFVKVKGTDQVLVK	....	TIKIIEFGQAHQL
Ictidomys_tridecemlineatus	CVETSSKKTFMAKFVKVKGTDQVLVK	....	TIKIIEFGQARQL
Latimeria_chalumnae	CVENTSKKTYMAKFVKVKGADQVLVK	....	LVKIIELGQARQL
Loxodonta_africana	CVETSSKKTFMAKFVKVKGTDQVLVK	....	TVKIIEFGQARQL
Macaca_mulatta	CVETSSKKTYMAKFVKVKGTDQVLVK	....	TIKIIEFGQARQL
Macropus_eugenii	CVETSSKKTYMAKFVKVKGTDQVLVK	....	VIKIIEFGQARQL
Meleagris_gallopavo	CVEAVSKKTYLAKFVKVKGADQVLVK	....	VVKIVEFGQARQL
Microcebus_murinus	CVETSSKKTYMAKFVKVKGTDQVLVK	....	TIKIIEFGQARQL
Monodelphis_domestica	CVETSSKKTYMAKFVKVKGTDQVLVK	....	TIKIIEFGQARQL
Mus_musculus	CVETSSKRTFMAKFVKVKGTDQVLVK	....	TIKIIEFGQARQL
Myotis_lucifugus	CVETSSKKTYMAKFVKVKGTDQVLVK	....	VIKIIEFGQARQL
Nomascus_leucogenys	CVETSSKKTYMAKFVKVKGTDQVLVK	....	TIKIIEFGQARQL
Ochotona_princeps	CVETSSKKTFMAKFVKVKGTDQVLIK	....	VIKIIEFGQARQL
Oreochromis_niloticus	CVEISSEKTYMAKFVKVRGADQTLVK	....	NVKIIELGQSRHL
Ornithorhynchus_anatinus	CVETSSKKTYMAKFVKVKGTDQVLVK	....	TIKIIEFGQARQL
Oryctolagus_cuniculus	CVETSSKKTFMAKFVKVKGTDQVLVK	....	VIKIIEFGQARQL
Oryzias_latipes_A	CTEIATKKTFMAKFIKVKGTDRELVL	....	NIKIIDMGQSRLL
Oryzias_latipes_B	CVNISSEKTYMAKFVKVRGADQAIIK	....	NVKIIELGQSRHL
Otolemur_garnettii	CVETSSKKTYMAKFVKVKGTDQVLVK	....	TIKIIEFGQARQL
Pelodiscus_sinensis	CVETVSKKTFLAKFVKVKGADQVLVK	....	IIKIIEFGQARQL
Petromyzon_marinus	CVEISSKKTYMAKFAKVKGADQGSTK	....	RVKLVEFGQARIL
Pongo_abelii	CVETSSKKTYMAKFVKVKGTDQVLVK	....	TIKIIEFGQARQL
Procavia_capensis	CVETSSKKTYMAKFVKVKGTDQVLVK	....	TIKIIEFGQARQL
Pteropus_vampyrus	CVETSSKKTYMAKFVKVKGTDQVLVK	....	TIKIIEFGQARQL
Rattus_norvegicus	CVETSSKKTFMAKFVKVKGTDQVLVK	....	IIKIIEFGQARQL
Sarcophilus_harrisii	CVETSSKKTYMAKFVKVKGTDQVLVK	....	VIKIIEFGQARQL
Sorex_araneus	CVETSSKKTYMAKFVKVKGTDQVLVK	....	LIKIIEFGQARQL
Sus_scrofa	CVETSSKKTYMAKFVKVKGADQVLVK	....	TIKIIEFGQARQL
Takifugu_rubripes_A	CVEIATKKTFMAKFIKVKGTDRELVL	....	NIKIIEMGQARLL
Takifugu_rubripes_B	CVDICSEKTYMAKFVKVRGADQALVK	....	NVKIIELGQCRHL
Tetraodon_nigroviridis_A	CVEIATKRTFMAKFIKVKGTDRELVL	....	TIKIIEMGQARLL
Tupaia_belangeri	CVETSSKKTYMAKFVKVKGTDQVLVK	....	TIKIIEFGQARQL
Xenopus_tropicalis	CIENSSKKTYLAKFVKVKGADQVLVK	....	TIKITEFGQARQL
Cricetulus_griseus	CVETSSKKTFMAKFVKVKGTDQVLVK	....	TIKIIEFGQARQL
Mustela_putorius_furo	CVETSSKKTYMAKFVKVKGTDQVLVK	....	TIKIIEFGQARQL
Heterocephalus_glaber	CVETSSKKTFMAKFVKVKGTDQVLVK	....	IIKIIEFGQARQL
Xiphophorus_maculatus_A	CVEIATKKTFMAKFIKVKGTDRELVL	....	NIKMIEMGQSRLL
Xiphophorus_maculatus_B	CVDISSEKTYMAKFVKVRGADQAIIK	....	TVKIIELGQSRHL

Invertebrate titin-like kinases (include twitchin, projectin and TTN-1 kinases)

```
Caenorabditis_elegans          NNFAAKFVM .... ELKLIDFGLTAHL
Caenorhabditis_briggsae        NNFAAKFVM .... ELKLIDFGLTAHL
Caenorhabditis_remanei         NNFAAKFVM .... ELKLIDFGLTAHL
Ascaris_suum                   NTFAAKFVN .... QLKLIDFGLAAKL
Brugia_malayi                  NTFAAKFVN .... QLKLIDFGLAAKL
Loa_loa                        NTFAAKFVN .... QLKLIDFGLAAKL
Trichinella_spiralis           NVFAAKFVN .... VLKLIDFGLAAKL
Tribolium_castaneum            NIFAAKFIP .... NIKLIDFGLATKL
Pediculus_humanus              SIFAAKFIP .... NIKLIDFGLATKL
Procambarus_clarkii            NIFAAKFIP .... NVKLIDFGLATKL
Harpegnathos_saltator          NIFAAKFIP .... NVKLIDFGLATKL
Mytilus_galloprovincialis      RVFVAKFIN .... EVKMIDFGLATKL
Crassostrea_gigas              RVFVAKFIN .... NVKMIDFGLATKL
Aplysia californica            RVFVAKFIN .... SVKIIDFGLATKL
Drosophila_willstoni           NIFAAKFIP .... SVKLIDFGLATRL
Drosophila_pseudoobscura       NIFAAKFIP .... NVKLIDFGLATRL
Drosophila_persimilis          NIFAAKFIP .... NVKLIDFGLATRL
Drosophila_melanogaster        NIFAAKFIP .... NVKLIDFGLATRL
Aedes_aegypti                  NVFAAKFIP .... NVKLIDFGLATRL
Anopheles_darlingi             NVFAAKFIP .... NVKLIDFGLATRL
Anopheles_gambiae              NVFAAKFIP .... NVKLIDFGLATRL
Culex_quinquefasciatus         NVFAAKFIP .... NVKLIDFGLATRL
```

Section S6: Modelling of the hypothesized active conformations of the catalytic domains of twitchin and titin kinases

The crystal structures of TK (PDB: 1tki) and TwcK (PDB: 1kob) kinases were used in this study. The TwcK structure is that from *Aplysia* as this contains a valine residue (instead of alanine) in position 2 of the VAIK motif. This choice was aimed to confirm through modelling the experimental observation that valine at this position does not interfere with ATP-binding and, therefore, catalysis. A BLAST (3) search of the TK and TwcK sequences against the PDB databank showed that the closest available relative in a closed conformation and complexed to ATP/Mg^{2+} was death-associated protein kinase 1 (DAPK) (PDB: 1jkl). DAPK shares 38% sequence identity with TK and 41% with TwcK (similarity 61% and 62%, respectively). Thus, DAPK was used here as a template to model the predicted closed conformations of TK and TwcK. Flanking residues were removed from the crystal structures of target and templates to obtain the core kinase domains, so that the CRD tails were not present in the models (models included residues 53-308 in 1KOB and 24-278 in 1TKI; residue numbering as in the respective PDB entries). TK and TwcK were divided into two sequential lobes (split points, 1kob: S131-G132; 1tki: S101-G102, numbering as in the corresponding crystals structures) and each lobe individually rigid-body fitted onto their DAPK counterparts using PyMOL v.1.3 (www.pymol.org). The repositioned lobes were re-annealed using Modeller v9.12 (4). A satisfactory model of TK was obtained in this way. The TwcK model required in addition the slight repositioning of αC-helix (helix H8 in the TK-like kinase family nomenclature) and residue F64 in the Glycine-rich loop to achieve optimal placement respect to the ATP ligand. The repositioning of αC-helix (slight lowering) was performed in Modeller by homology to DAPK (and comparatively assessed against TK). Models were checked for Ramachandran plot quality using RAMPAGE (5): the final models contain no residues in outlier regions.

Fig S6: Open- and closed-lobe conformations of TK and TwcK kinases
Upper: Unbound, open lobe conformations observed in crystals structures. In TwcK and TK, the CRD segment has been removed. (Lower) ATP-bound, closed-lobe conformations modelled using entry 1JKL as template. The ATP molecule and magnesium atoms (green) are displayed as corresponding to the superposition of DAPK (1JKL) and the models calculated. Residues flanking the adenine pocket are shown (purple) and labelled. TwcK shows a regularly shaped ATP-binding pocket with common molecular features, validating the modeling approach. In TK, the ATP-binding pocket does not appear well formed; M34 contributes to that divergence. The M34 rotamer displayed was the only geometry that did not result in clashes with neighbouring TK side chains. (PDB codes are given for experimental structures).

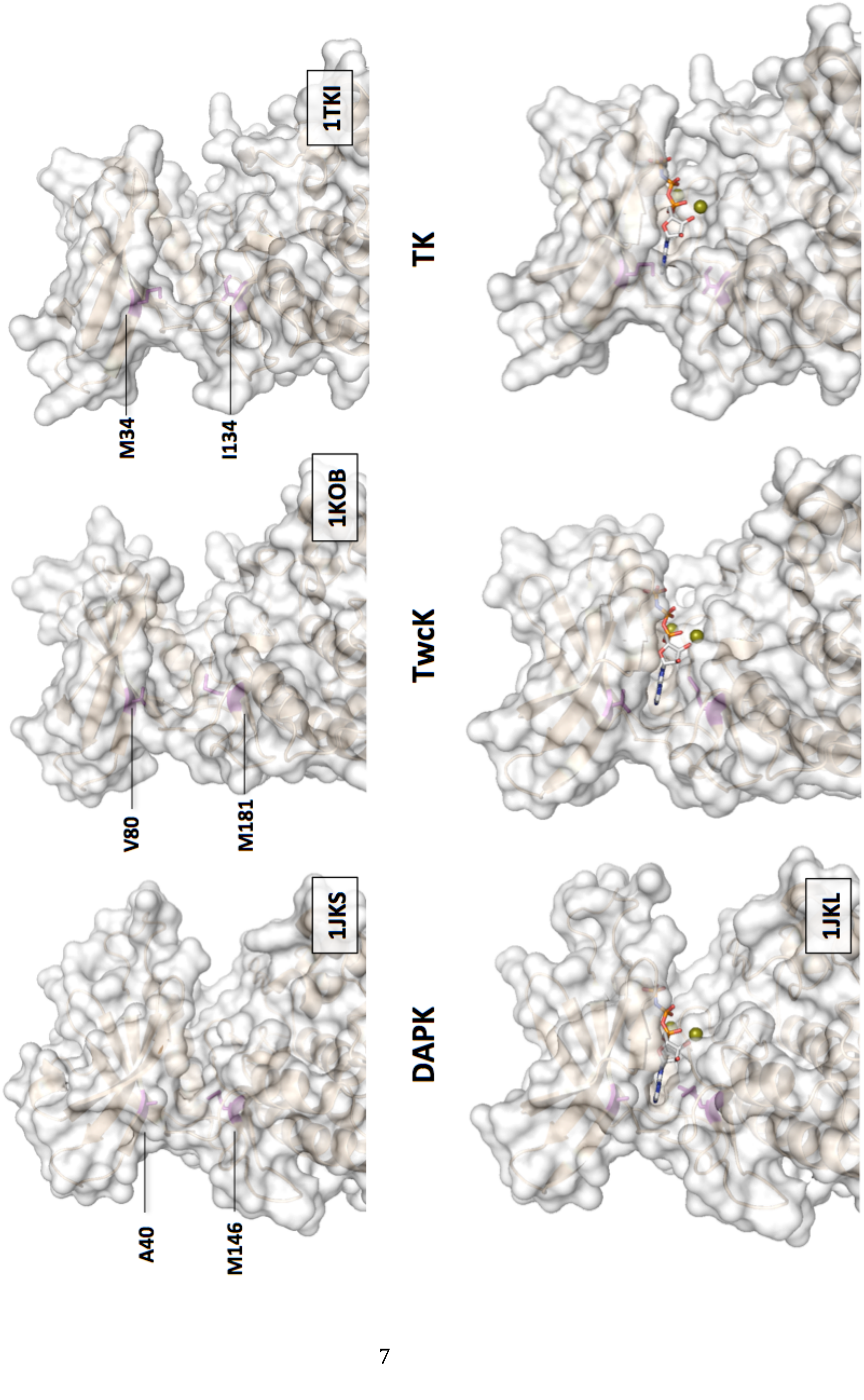
DAPK
TwcK
TK
1JKS
1KOB
1TKI
1JKL
A40
M146
V80
M181
M34
I134

References

1. Van Durme, J., Delgado, J., Stricher, F., Serrano, L., Schymkowitz, J., Rousseau, F. 2011 A graphical interface for the FoldX forcefield. *Bioinformatics* **27**, 1711-1712.

2. Matulis, D., Kranz, J.K., Salemme, F.R., Todd, M.J. 2005 Thermodynamic stability of carbonic anhydrase: measurements of binding affinity and stoichiometry using ThermoFluor. *Biochemistry* **44**, 5258-5266.

3. Altschul, S.F., Gish, W., Miller, W., Myers, E.W., Lipman, D.J. 1990 Basic local alignment search tool. *J Mol Biol.* **215**, 403-410.

4. Sali, A., Blundell, T.L. 1993 Comparative protein modelling by satisfaction of spatial restraints. J Mol Biol **234**, 779-815.

5. Lovell, S.C., Davis, I.W., Arendall III, W.B., de Bakker, P.I.W., Word, J.M., Prisant, M.G., Richardson, J.S., Richardson, D.C. 2002 Structure validation by Calpha geometry: p hi, psi and Cbeta deviation. Prots: Struct, Func & Gen **50**, 437-450.
